# Favorable results from a prospective evaluation of 200 patients with large hiatal hernias undergoing LINX magnetic sphincter augmentation

**DOI:** 10.1007/s00464-017-5859-4

**Published:** 2017-09-21

**Authors:** F. P. Buckley, Reginald C. W. Bell, Kate Freeman, Stephanie Doggett, Rachel Heidrick

**Affiliations:** 1 The Heartburn & Acid Reflux Center, Baylor Scott & White Healthcare, Round Rock, TX USA; 2 SurgOne Foregut Institute, 401 W Hampden Place Suite 230, Englewood, CO 80110 USA

**Keywords:** GERD, LINX, MSA, Magnetic sphincter augmentation, Hiatal hernia, Paraesophageal hernia

## Abstract

**Introduction:**

Magnetic sphincter augmentation (MSA) of the lower esophageal sphincter restores the antireflux barrier in patients with hiatal hernias ≤3 cm. We performed a prospective study in patients undergoing MSA with the LINX device during repair of paraesophageal and hernias over 3 cm axial component.

**Methods and procedures:**

Multicenter, prospective study of consecutive patients treated with MSA at the time of repair of hiatal hernias >3 cm.

**Results:**

200 patients (110 female) were treated between March 2014 and February 2017 via laparoscopic hernia repair and MSA. Mean age was 59.5 years, mean BMI 29.4. 40% had esophagitis, 20% intestinal metaplasia, 72 of 77 tested had abnormal pH studies. Preoperative PPI use was reported by 87%. Eighteen patients had prior hiatal hernia/fundoplication. All had normal function. 78% of patients had axial hiatal hernia ≥5 cm or large paraesophageal component. Mean operative time was 81 min (38–193), EBL was 10 cc. Non-permanent mesh reinforcement of hiatal repair was performed in 83% of the patients. There were two readmissions for dehydration; 2 patients with pulmonary embolism, and 1 patient with cardiac ischemia. Nineteen patients required dilation. 156 pts were followed at a median of 8.6 months. GERD-HRQL scores improved from 26 preoperatively to 2 postoperatively. Complete PPI independence was achieved in 94% (147/156). Videoesophagram in 51 patients at median 11 months found 3 asymptomatic hernias <3 cm. One symptomatic patient underwent successful repair of the hernia without MSA manipulation. There have been no device explants, erosions, or migrations to date.

**Conclusions:**

This prospective study of 200 patients with >3 cm hernias undergoing MSA with hiatoplasty resulted in favorable outcomes with median of 9 months follow-up. Comparing this to published reports of MSA in patients with <3 cm hernias, the safety and clinical efficacy of MSA are independent of initial hernia size.

Gastroesophageal reflux disease (GERD) is one of the most common diseases encountered by clinicians and its prevalence is estimated to be as high as 20–30% in westernized countries [1, 2]. GERD results from the failure of the antireflux barrier, commonly a defective lower esophageal sphincter, which allows for abnormal reflux of gastric contents into the esophagus. First-line therapy for GERD with medical treatment leaves up to 40% of patients with incomplete resolution of symptoms, as medicine alone does not address the mechanical pathophysiology of the disease [3]. Nissen fundoplication has long been considered the gold standard antireflux operation. Despite its well-established long-term efficacy, it is estimated that antireflux procedures are performed in less than 1% of patients who have failed medical treatment. The reasons for this are multifactorial and include lack of reproducibility and potential side effects. [1].

Magnetic sphincter augmentation (MSA) with the LINX^®^ Reflux Management System (Torax Medical, Maple Grove, MN) was approved in 2012 by the FDA. Numerous studies have shown MSA to be safe and effective in the treatment of GERD and an excellent alternative to the classic Nissen fundoplication. [4, 5] Advantages of MSA over a Nissen fundoplication include improved side-effect profile, minimal disruption of anatomy, and reproducibility. [6] MSA augments the resting lower esophageal sphincter pressure, prevents effacement, and reduces gastric reflux. In contrast to the static nature of a fundoplication, the ability of MSA to open dynamically allows for more physiologic transport of food boluses, vomiting, and belching.

Initial studies of the device excluded patients with hiatal hernias greater than 3 cm, and FDA approval of the device considered use in these patients a ‘precaution.’

Recent high-resolution manometry studies have confirmed earlier literature demonstrating that the crural diaphragm contributes significantly to the antireflux mechanism. Crural repair and fundoplication both contribute to an increase in the high-pressure zone [7]. The efficacy of the repaired crural diaphragm in preventing reflux may be similar regardless of the initial hernia size.

As the size of a hiatal hernia increases, indications for surgery begin to include symptoms and problems related to the hernia itself, e.g., obstruction and strangulation. The surgical management of these large hernias frequently entails a fundoplication both to prevent reflux and reherniation by creating a ‘buttress.’ [8–10] Post-fundoplication symptoms can be particularly troublesome in patients who did not initially present with GI symptoms.

Use of MSA may be a viable substitute for a traditional fundoplication in patients with larger hiatal and paraesophageal hernias. Once the crural component of the antireflux barrier is re-established, augmentation of the LES may be sufficient to complete the antireflux mechanism, and while avoiding the troublesome side-effect profile of fundoplication. Additionally, the MSA device becomes fixed in place around the distal esophagus by capsular formation fairly quickly after surgery [11]. The collar created by the MSA may have a role as a buttress to mitigate against reherniation.

One retrospective study has reported that patients undergoing MSA had similar results in patients with hernias over 3 cm as in patients with hernias ≤3 cm [12].

Our study prospectively evaluates the clinical effectiveness of MSA in patients with larger hernias, including those with paraesophageal hernias, in whom an antireflux procedure would be performed routinely after herniorrhaphy.

## Study design and patients

A prospective multicenter community study was approved by the Institutional Review Board at each site. Patients provided written informed consent. Patients who would be undergoing hiatal hernia repair with a concomitant antireflux procedure were evaluated for the study. Entry criteria included a hiatal hernia >3 cm by preoperative endoscopy, barium swallow, or CT scan. Patients undergoing revisional surgery after primary laparoscopic fundoplication were considered eligible if other criteria were met. Patients were of an age and ability to provide informed consent.

Duration of symptoms, as well as duration, daily use of, and response to acid-suppressive medication were recorded. All patients responded to a standardized set of quality-of-life questions (GERD-HRQL). Site specific questions on regurgitation and laryngopharyngeal symptoms were also recorded. Preoperative evaluation included evaluation of esophageal clearance by esophageal manometry and/or semi-solid bolus video esophagram. Patients undergoing surgery for a primary reason of GERD underwent ambulatory reflux testing if other objective measures of GERD were lacking. Patients undergoing surgery for a primary reason of hiatal hernia did not routinely undergo preoperative reflux testing.

## Methods

Surgery was performed by a single surgeon at each of the two sites (FPB and RB). Patients underwent routine dissection of the hernia sac and mediastinum until adequate esophageal length was obtained. Cruroplasty was performed with permanent suture until the hiatus was gently brought into apposition with the relaxed esophagus. Reinforcement of the hiatal repair was performed with a non-permanent prosthetic mesh if deemed appropriate by the individual surgeon. The posterior vagus was elevated off the posterior esophagus and the MSA sizer was introduced. The sizer was closed until it rested smoothly but non-compressively against the relaxed, non-distended esophagus. An MSA with a corresponding number of beads was then placed between the posterior esophagus and posterior vagus, reapproximated anteriorly, and the clasp actuated. The MSA was positioned above the angle of His in all instances and preferably the device was placed cephalad to an intact first gastric branch of the posterior vagus. If the posterior vagus had been elevated for some distance, as occasionally occurred during dissection of a large hernia sac, a small polypropylene suture was placed from distal esophagus to perineurium of the posterior vagus distal to the MSA device to provide posterior anchoring. Patients were discharged when they met routine postoperative requirements. Intraoperative and 30 day complications were recorded. Diet was advanced as for routine MSA procedures.

Patients responded to the same QOL questions used preoperatively. Patients were asked to undergo postoperative barium swallow or endoscopy/pH testing between 6 months and 1 year postoperatively. Each site performed its own follow-up.

Primary endpoints evaluated in this study included (1) safety of the procedure, (2) improvements in Quality-of-Life Assessments, and (3) ability to achieve independence from daily proton-pump inhibitor medication use. Secondary endpoints were objective recurrence of hernia or reflux measured by barium swallow or endoscopy/pH test.

Data were collected prospectively at each site and then combined for analysis after removing any patient identifiers. Parametric and non-parametric statistics (*T* test, Mann–Whitney U) were used as appropriate to describe and compare data.

## Results

### Patient characteristics

A total of 200 patients (110 female, 90 male) were treated between March 2014 and February 2017 via laparoscopic hiatal hernia repair and MSA. Mean age was 60 years (range 21–93), Mean BMI 29 kg/m^2^ (range 19–49). All patients presented with a hiatal hernia over 3 cm by endoscopy or radiographic imaging.

Seventy-eight percent presented with a hiatal hernia of 5 cm or greater, and 29% presented with an intrathoracic stomach (≥50% or ≥10 cm hernia). Mean axial hernia height by endoscopy was 4.2 cm and mean greatest cranio-caudal dimension by esophagram was 6 cm. Seventeen (8.5%) had undergone a prior hiatal hernia repair with fundoplication.

Twenty percent of patients had Barrett’s metaplasia, and 40% esophagitis. Patients with esophagitis, Barrett’s esophagus, or with hiatal hernia as primary indication for surgery did not routinely undergo ambulatory reflux testing. Excess reflux was demonstrated in 72 (96%) of the 77 patients who did undergo preoperative ambulatory reflux testing. Eight-five percent of patients had GERD documented by esophagitis, Barrett’s esophagus, abnormal pH, and/or PPI use.

All 200 patients were considered to have normal esophageal body function by objective evaluation. Of 121 tested, 107 had normal solid bolus transit by video esophagram (VEG), and 115 of 116 tested had normal esophageal manometry findings. The remaining patient demonstrated mildly elevated LES residual pressure on manometry, had no dysphagia and normal solid bolus transit by VEG, and was considered appropriate for MSA.

All patients had either GERD or symptomatic hiatal hernia (or both) as the indication for surgery. Forty-four patients (22%) presented with a hiatal hernia between 3 and 5 cm. All of these patients had GERD as the primary indication for surgery. Symptomatic hiatal hernia was the primary indication for surgery in the majority of the 156 patients with a ≥5 cm hiatal hernia, though many of these patients had significant GERD symptoms as well. Symptoms and acid-suppressive medication use are therefore reported for the aggregate group of 200 patients. Median preoperative GERD-HRQL was 26 (0–50). Dysphagia was present in 28% of patients, and regurgitation in 61% of patients. Seventy percent of patients reported laryngopharyngeal reflux symptoms (LPR). The median reflux symptom index (RSI), a measure of LPR symptoms, was 17 (0–50) [13]. Routine preoperative PPI use was reported by 87% of patients.

### Operative findings

All procedures were completed with laparoscopic technique. Mean operative time was 81 min (38–193). Median estimated blood loss was 10 cc and <100 cc in all.

Extensive (>7 cm, typically up to the inferior pulmonary vein) esophageal mobilization was required in 65% (67 of 103 cases where recorded), including mobilization of posterior and/or anterior vagus nerve in 29 patients. With extensive mobilization, no patient required a Collis gastroplasty. At least 2 cm of intraabdominal esophagus (measured to the angle of His) was obtained in all patients.

Mean hiatal dimensions in 103 patients were 5.5 cm anterior–posterior by 3.2 cm transverse (Range 3–10 AP, 1.8–7 transverse). Hiatal Surface Area was 8.1 cm^2^ (2–24). Suture technique was simple 0-Ethibond in 95 and simple 0-Ethibond with 5 mm pledgets in 105 patients. Non-permanent bioabsorbable mesh was used to reinforce a primary hernia repair in 83% of patients. No patient required a relaxing incision or bridging with mesh.

An MSA device was placed between the posterior vagus and the esophagus in all patients. Sixteen patients with a mobilized posterior vagus had a polypropylene suture placed anchoring the esophagus to the posterior vagus perineurium, thus creating a caudal delimiter for the MSA. Median MSA size was 15 beads (range 13–17 beads).

### Safety

The MSA implantation in 200 patients with >3 cm hiatal hernias resulted in no major perioperative complications. Two readmissions within 30 days for dysphagia/dehydration were probably related to the procedure. Three other 30-day complications (2 pulmonary embolism and 1 patient with cardiac ischemia) were related to the complexity of the procedure or underlying cardiac risk factors and were not attributable to the MSA implantation per se.

Nineteen patients (10%) have undergone postoperative dilations (median 1, maximum 3 dilations) with improvement or resolution of the dysphagia. Two patients (1%) have undergone reoperation. One patient developed chest pain 6 months postoperatively; esophagram demonstrated the MSA migrating to and fro about the hiatus; laparoscopic surgery to reclose the hiatus above the MSA was successfully performed and the patient is doing well 9 months postoperatively. One patient with persistent reflux and aspiration after the MSA underwent successful conversion to fundoplication. There have been no device erosions or migrations to date.

### Symptomatic outcomes

**Fig. 1:**
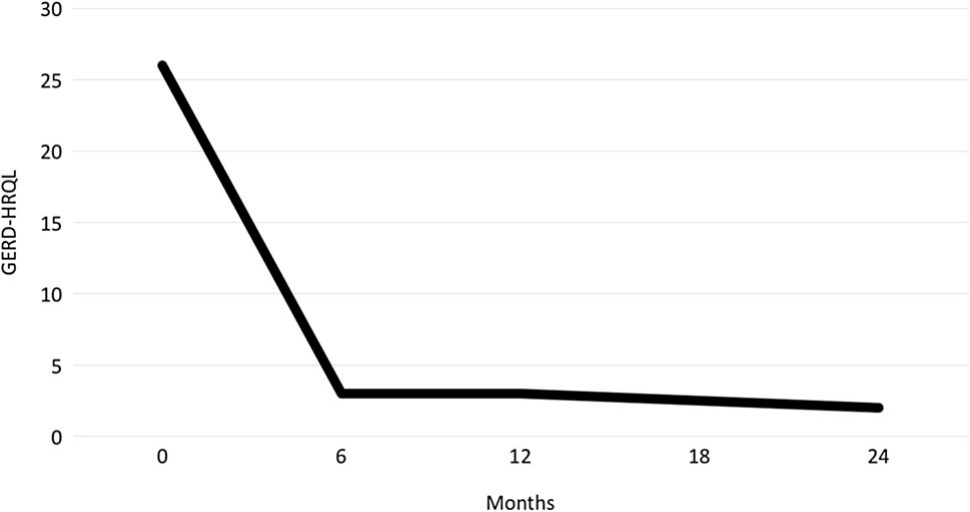
Median GERD-HRQL Scores. Numbers of patients completing the survey are 192 preoperatively, 102 at 6 months, 63 at 12 months, and 15 at 24 months

Median GERD-HRQL score was 2 (0–24) at a median follow-up of 258 (30–1058) days. GERD-HRQL scores at 6, 12, and 24 months remained stable (Fig. 1).

**Fig. 2:**
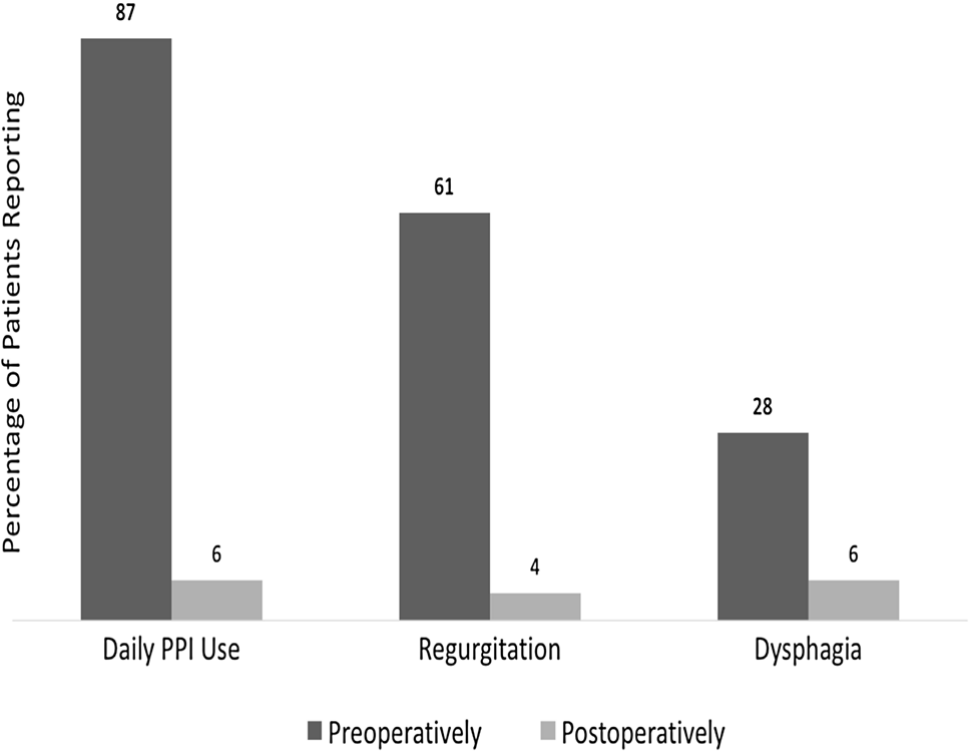
Percentage of patients reporting daily PPI use, regurgitation, and dysphagia before and after surgery

Similar improvements were seen in the RSI (17 preoperatively to 1 postoperatively). Regurgitation symptoms were reported by 61% preoperatively, 4% at 6 months, and 5% at 12 months. Dysphagia, present in 28% of patients preoperatively, was present in 6% of patients at last follow-up (Fig. 2).

### Objective outcomes

Routine postoperative esophagram requested of patients 9–12 months after surgery was performed on 51 of 80 eligible patients (64%) at a median of 11 months. No patient had a recurrent hernia of over 2 cm. Four (16%) patients were noted to have a supradiaphragmatic LINX, all ≤2 cm axially. Three were asymptomatic; the other patient had reoperation as noted above. The one patient with LA D esophagitis preoperatively had LA C esophagitis at follow-up endoscopy; though asymptomatic, she was placed on acid-suppressive medication.

Independence from daily PPI use was achieved in 147 of 156 (94%) of patients.

## Discussion

Magnetic sphincter augmentation dynamically augments the LES, preventing pathologic reflux while preserving gastroesophageal anatomy [11]. Initial studies of the LINX^®^ MSA system excluded patients with greater than 3 cm hernias [4, 14]. This study exclusion criteria carried over to the FDA approval and use of the LINX^®^ device in patients with >3 cm hernias is listed as a “precaution,” meaning simply that its safety and efficacy in patients with larger hernias had not been assessed. The Instructions for Use state “Use of LINX^®^ device in patients with a hiatal hernia larger than 3 cm should be considered on the basis of each patient’s medical history and severity of symptoms” [15]. This information is in the public domain and available on the FDA and manufacturer’s websites.

Initial studies left it up to the operating surgeon on whether to perform a “minimal dissection” or perform a crural dissection and cruroplasty. As outcomes were analyzed, crural repair in patients with hiatal hernias emerged as a factor in successful outcomes after MSA [16].

Rona and the USC group, in a retrospective analysis, were the first to report that use of MSA in patients with larger hiatal hernias resulted in favorable outcomes similar to those in patients with ≤3 cm hernias [12].

High-resolution manometry has confirmed the “two sphincter theory” that the LES and the crural diaphragm contribute independently to the antireflux barrier. [17–19]. Intraoperative manometry confirms that crural repair contributes to the postoperative high-pressure zone. [7, 20]. Appropriate reconstruction of the crural diaphragm corrects one of the two defects in the reflux barrier.

We hypothesized that reconstruction of the crural diaphragm could successfully be combined with augmentation of the LES with the MSA device regardless of the initial size of the hiatal defect, and we set out prospectively to study patients with over 3 cm hiatal hernias.

Patients with large or paraesophageal hernias are frequently treated with an antireflux procedure at the time of hernia repair regardless of preoperative reflux symptoms, both to help prevent recurrence of the hernia and to mitigate the development of de novo reflux [9]. An antireflux procedure has been associated with a lower hernia recurrence rate [8]. The postulated reason is that the bulk of the fundoplication creates a buttress around the esophagus, helping to fix the esophagus in the abdomen [21]. De novo reflux can develop after repair of a large hiatal hernia without fundoplication [8, 22]. The mechanism for de novo reflux is suggested by a study by Marchand in which removal of the left leaf of the diaphragm—as might occur during a paraesophageal hernia—increased the intragastric pressure required to induce reflux from 28 to 42 cm of water. Conversely, restoring the fundus to its normal position beneath the left hemidiaphragm might lead to incompetence of the reflux barrier [23]. Furnee found that patients without reflux prior to repair of a PEH developed either GERD symptoms (39%) or reflux esophagitis (28%) if no antireflux procedure was performed. A randomized controlled trial of repair of PEH with and without fundoplication found significantly higher postoperative DeMeester scores (40.9 vs 9.6), esophagitis (53 vs 17%), and reflux symptom scores (1.9 vs 1.1) in those patients not receiving fundoplication [24].

Based on this study, we believe that MSA can serve as an alternative to fundoplication in patients with large hiatal hernias, both to limit GERD and to create a collar that mitigates against reherniation of the stomach. We believe it appropriate to offer MSA in the absence of pH testing in these patients knowing that a certain percentage with normal pH testing preoperatively would be at risk for postoperative reflux [25, 26]. In fact, we believe the lower incidence of bloating and flatulence seen with MSA [27] may make it a better choice than a fundoplication especially in patients who did not have GERD symptoms prior to their surgery.

As outcomes of MSA had not been widely reported in patients with hernias >3 cm [12], a primary endpoint of the study was safety. The current study of patients with >3 cm hernias undergoing laparoscopic hernia repair and MSA placement included those with primarily GERD symptoms, patients with >5 cm hernias (PEH or otherwise) for hernia-related reasons, and patients undergoing revisional surgery after prior hiatal/reflux surgery. There were 2 device-related readmissions (1%) for dehydration/dysphagia, and no unanticipated or long-term device-related complications within the first 30 days. Recognizing that follow-up is still short-term, reoperation rates have been 1%, well within the 3% range reported in prior MSA studies [28]. No migrations or erosions have been observed.

It should be noted that the recurrent hernias we have observed after MSA have been short axial, transdiaphragmatic herniations in which the MSA device has maintained its proper position around the distal esophagus. Correction of this recurrent hernia after MSA is different from correction of a herniated fundoplication. The hiatus has been fairly easily dissected from the esophagus and encapsulated MSA and then dissection has proceeded up the mediastinum until adequate length is once again achieved. The hiatus has then been reapproximated. In comparison, a herniated fundoplication requires dissection of the fundoplication from the hiatus and often the esophagus, with risk of gastric, esophageal, or vagal injury. We have not observed significant mediastinal adhesions from a supradiaphragmatic MSA, nor have others [28].

**Fig. 3:**
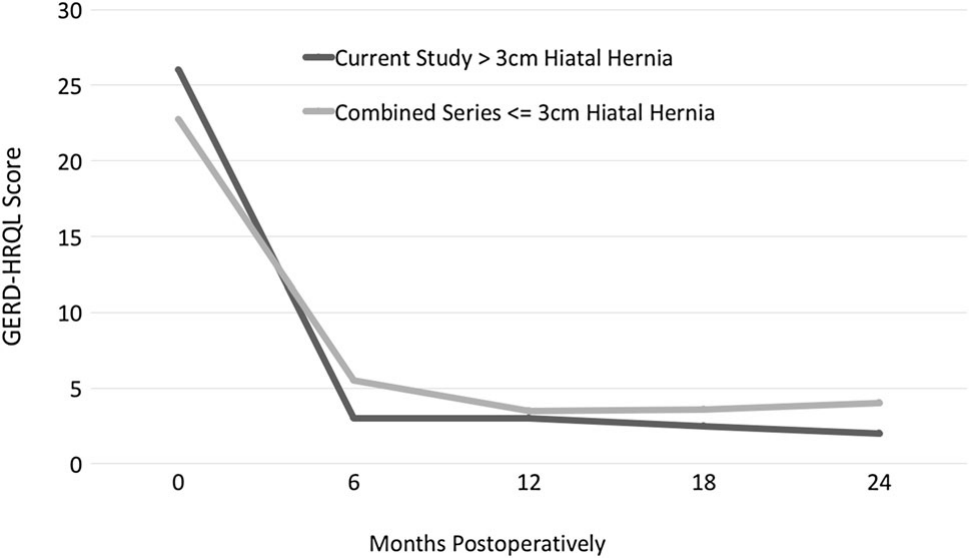
GERD-HRQL scores of current study in comparison to combined series [3, 12, 31–33]

Symptomatic outcome measures using validated quality-of-life surveys have been used in other studies of MSA and provide a standard against which to measure the outcomes of this study [4]. Symptomatic outcomes of patients at 6 months and 1 year with large hiatal hernias undergoing MSA demonstrated mean GERD-HRQL scores <5 postoperatively in all patients, as well as in that subgroup of patients who had elevated GERD-HRQL scores preoperatively. These results are comparable to patients with hernias ≤3 cm undergoing MSA [11, 29] (Fig. 3). Independence from daily PPI use was 94% among all patients, and 85% among patients using PPIs daily preoperatively.

Studies of laparoscopic PEH repairs with or without biologic mesh, using hernia size of over 2 cm as a measure of recurrence, have reported objective recurrence rates of 7–24% at 6 months [30, 31]. Both sites in this study used a non-permanent mesh as an overlay after primary cruroplasty. Objective outcome measurements obtained in 51/80 patients demonstrated no hernias over 2 cm in size at a median of 11 months postoperatively.

Studies of MSA in patients with ≤3 cm hernias have been remarkably consistent. GERD-HRQL scores have decreased from ~20 to less than 5. Independence from daily PPI use has been achieved in ~90% of patients. Hiatal hernia repair with mesh and MSA resulted in no higher dysphagia and dilation rates than other studies with MSA [11, 12]. Reoperation rates (typically for dysphagia or recurrent reflux) have been under 3% in most series [5, 28, 32]. The results of our current study with larger hiatal hernias are comparable to these other reports (Fig. 3).

The study had several limitations. Although all patients had greater than 3 cm axial component to the hernia by preoperative or intraoperative findings, no single measurement of hernia size was used uniformly. The two institutions performed slightly different prospective data collection and surgical techniques. We could be criticized for not obtaining preoperative pH testing on all patients; however, the false-negative rate seen in patients with large hernias limits the utility of pH testing [9]. We did not directly compare outcomes of patients in this study with outcomes of our patients with ≤3 cm hernias. Outcomes from patients undergoing MSA with ≤3 cm hernias have been reported elsewhere, are remarkably consistent, and we thought adequate to serve as a benchmark against which to compare this study’s results [3, 11, 29]. We would have preferred to obtain more complete objective postoperative data, especially on asymptomatic patients; this was not possible insofar as many asymptomatic patients refused objective testing for cost and/or travel reasons. We intend to continue reporting outcomes up to 5 years and will continue to collect as much objective data as possible.

As in other areas of medicine, initial research criteria can become *de facto* clinical criteria. Researchers studying Barrett’s esophagus excluded patients with <3 cm of columnar-lined esophagus in order to reduce false-positives in their findings. It awaited Spechler and associates to study patients with <3 cm CLE before short-segment Barrett’s was even recognized as an entity [33]. The initial MSA research criteria excluded patients with a hiatal hernia >3 cm. This study strongly supports that the benefit of MSA extends beyond patients with small hernias. The two sphincter theory explains why our results with larger hernias is equivalent to those reporting on smaller or no hernia. Crural closure, regardless of size, restores one element of the antireflux barrier, while MSA restores the LES, the other and independent component of the antireflux barrier. In addition, this study found a low hernia recurrence rate, suggesting that the ‘collar’ of the MSA device may provide buttress effect similar to that of fundoplication in repair of large hiatal hernias.

## Conclusions

This prospective study of 200 patients with >3 cm hernias undergoing MSA with hiatoplasty resulted in favorable outcomes at a median of 9 months follow-up. Comparing this large study to published reports of MSA in patients with <3 cm hernias, the safety and clinical efficacy of MSA are independent of initial hernia size.
